# Enhancement of HIFU ablation by sonosensitizer-loading liquid fluorocarbon nanoparticles with pre-targeting in a mouse model

**DOI:** 10.1038/s41598-019-43416-y

**Published:** 2019-05-06

**Authors:** Yong Zhang, Lijun Yong, Yong Luo, Xiaoya Ding, Die Xu, Xuan Gao, Sijing Yan, Qi Wang, Jie Luo, Darong Pu, Jianzhong Zou

**Affiliations:** 1 State Key Laboratory of Ultrasound Engineering in Medicine Co-Founded by Chongqing and the Ministry of Science and Technology, College of Biomedical Engineering, Chongqing Key Laboratory of Biomedical Engineering, Chongqing Medical University, Chongqing Collaborative Innovation Center for Minimally-invasive and Noninvasive Medicine, Chongqing, 400016 China; 2 Department of Ultrasound, the First Affiliated Hospital of Chongqing Medical University, Chongqing, 400042 China; 3 Department of Obstetrics, Chongqing Health Center for Women and Children, Chongqing, 401147 China; 4 Department of Ultrasound, Chongqing Traditional Chinese Medicine Hospital, Chongqing, 400021 China

**Keywords:** Targeted therapies, Outcomes research

## Abstract

High intensity focused ultrasound (HIFU) is a noninvasive thermal ablation technique for the treatment of benign and malignant solid masses. To improve the efficacy of HIFU ablation, we developed poly (lactide-co-glycolide) (PLGA) nanoparticles encapsulating perfluoropentane (PFP) and hematoporphyrin monomethyl ether (HMME) as synergistic agents (HMME+PFP/PLGA). Two-step biotin-avidin pre-targeting technique was applied for the HIFU ablation. We further modified the nanoparticles with streptavidin (HMME+PFP/PLGA-SA). HMME+PFP/PLGA-SA were highly dispersed with spherical morphology (477.8 ± 81.8 nm in diameter). The encapsulation efficiency of HMME and PFP were 46.6 ± 3.3% and 40.1 ± 2.6%, respectively. The binding efficiency of nanoparticles to streptavidin was 95.5 ± 2.5%. The targeting ability of the HMME+PFP/PLGA-SA nanoparticles was tested by parallel plate flow chamber *in vitro*. In the pre-targeting group (HMME+PFP/PLGA-SA), a large number of nanoparticles bound to the peripheral and surface of the cell. In the HIFU ablation experiment *in vivo*, compared with the other groups, the largest gray-scale changes and coagulation necrosis areas were observed in the pre-targeting (HMME+PFP/PLGA-SA) group, with the lowest energy efficiency factor value. Moreover, the microvessel density and proliferation index declined, while the apoptotic index increased, in the tumor tissue surrounding the coagulation necrosis area in the pre-targeting group. Meanwhile, the survival time of the tumor-bearing nude mice in the pre-targeting group was significantly longer than that in the HIFU treatment group. These results suggest that HMME+PFP/PLGA-SA have high potential to act as synergistic agents in HIFU ablation.

## Introduction

High-intensity focused ultrasound (HIFU) is a rapidly developing technique for microinvasive ablation of tumors in recent years. The therapeutic principle of HIFU is to focus multiple low-energy ultrasonic beams on the target regions in a certain way, inducing the thermal, cavitation, and mechanical effects to cause irreversible coagulation necrosis and eventual lesion resection^1^. At present, satisfactory results have been obtained for the clinical treatment of liver cancer, prostate cancer, pancreatic cancer, osteosarcoma, and uterine fibroids^2^. However, the ultrasonic energy would be attenuated with the increased propagation distance^3^, and the heat would be absorbed by the blood flow^4^. In addition, any bone or gas barrier along the acoustic beam path would also weaken the energy accumulation in the target area^5,6^. For the treatment of deep tumors or tumors with barrier along the acoustic beam path, if only increasing the output power of HIFU to deposit enough energy in the target area, it would bring great risk of damage to the normal tissues along the acoustic beam path^7^. Therefore, it is important to find a way to increase the efficiency of HIFU ablation and minimize damages to surrounding normal tissue.

For the above reasons, various synergistic agents have been tried, such as lipiodol, hydroxyapatite, ultrasound microbubbles, liquid fluorocarbon nanoparticles, mesoporous silica, and sonosensitizers^8–10^. It is desirable to find a synergistic agent that can effectively change the tissue acoustic environment and efficiently ablate the tumor tissue without injuring the surrounding normal tissues. One of the most commonly used synergistic agents would be the ultrasound microbubbles^11^, however, its micron-level size only allows for the circulation within the tumor microvasculature. Thus, ultrasound microbubbles cannot enter the tumor extravascular space. Meanwhile, the clinical application of ultrasound microbubbles is limited, due to its poor stability, short half-life *in vivo*, difficulties in cavitation control, and tissue damage along the beam path^12^. At present, liquid fluorocarbon nanoparticles, prepared by encapsulating liquid fluorocarbon with lipid or polymer materials, are considered as potent synergistic agents because their nano-size and liquid-gas phase transition^13^. They are liquid at room temperature, when temperature rises or there is ultrasonic irradiation, the liquid fluorocarbon in the nanoparticles would change from liquid to gas and form microbubbles, thereby increasing the cavitation effect of HIFU treatment^14–17^. They can also be used for ultrasound imaging and therapy^18,19^. When designing the phase-change synergistic agents, the liquid fluorocarbon comprising the core would create inherent trade-offs. For the HIFU enhanced ablation, nanoparticles need to be stable at body temperature, and phase transition would occur at lower temperatures without obvious side effects on surrounding tissues. Currently, two kinds of liquid fluorocarbons have been well studied, i.e., the perfluorohexane (PFH) and perfluoropentane (PFP) (with the boiling points of 56 °C and 29 °C, respectively). Other studies on fluorocarbon nanoparticles are relatively few because their boiling points are either too high or too low (PFB, 2 °C; OFP, −37 °C; and PFCE, 146 °C)^20,21^. Previous studies have shown that the phase transition temperature for the liquid fluorocarbon encapsulated in microspheres or nanoparticles is higher than that in the free status^22–25^. The phase transition temperatures for PFP and PFH microspheres/nanoparticles range from 37 °C to 45 °C and from 68 °C to 71 °C, respectively. Differential phase transition temperatures might be due to the different materials, encapsulating methods, and particle sizes. Zhou *et al*.^26^ have produced lipid nanoparticles encapsulating PFH and used them as the synergistic agents for HIFU. Their results showed significantly enhanced HIFU ablation, both *in vivo* and *in vitro*. This might be caused by the phase transition of PFH induced by the high temperature at the focal point of HIFU, which produces bubbles to change the acoustic environment and increase the energy accumulation. However, the phase transition temperature of the nanoparticles encapsulating PFH is above 60 °C, and this temperature is high enough to induce coagulation necrosis in the tissue. On the other hand, the phase transition temperature for PFP macromolecule nanoparticles is generally from 42 °C to 45 °C, which ensures the stability at body temperature and allows for the phase change at relatively low temperature. Therefore, PFP was selected as phase transition material to enhance the HIFU ablation in our study.

Sonodynamic therapy (SDT) is a novel tumor therapeutic method that involves a combination of low-intensity ultrasound and sonosensitizer. Essentially, both aspects (the sonosensitizer and ultrasound exposure) are harmless, however, cytotoxic events occur when they are combined. Hematoporphyrin monomethyl ether (HMME) is a kind of monomeric porphyrin purified from hematoporphyrin, which is characterized by simple composition, good selectivity, and shortened dark time after treatment^27^. It can be used for the treatment of various diseases, such as skin diseases, liver cancer, breast cancer, hematological tumors, and gliomas^27^. However, HMME is relatively hydrophobic and tends to clump in water, which affects its bioavailability. Fortunately, nanoparticle technology has great potential to address many shortcomings, such as increased SDT efficacy, binding avidity, and targeting specificity. In a previous study, HMME/PLGA (poly (lactic-co-glycolic acid) nanoparticles enhanced the HIFU ablation effects on ovarian cancer in nude mice with SDT^28^. In addition, some scholars have used nanoparticles loaded with chemotherapeutic drugs for HIFU synergistic ablation, but it is uncertain whether the chemotherapeutic drugs can maintain their original activity under high temperature and high pressure at HIFU focus^29,30^. The synergistic therapy of sonodynamic therapy and HIFU ablation is feasible in theory. Our aim is to combine liquid fluorocarbon nanoparticles with sonosensitizer to prepare a new nanoparticle. We hypothesize that this new nanoparticle would undergo the liquid-gas phase change under the HIFU irradiation to generate microbubbles. The microbubbles could not only change the tissue acoustic environment, but also increase the cavitation effect and activate the sonosensitizer efficacy when disrupted, thus achieving the synergistic effects of HIFU ablation and the sonodynamic therapy.

For targeting, synergistic agents are modified to be conjugated with antibodies or other ligands^31–33^. Tang *et al*.^29^ and Zhang *et al*.^30^ have prepared targeted nanoparticles by folate (PFH/DOX@PLGA/Fe3O4-FA) and HLA antibody (mAbHLA-G/MTX/PLGA) for HIFU ablation, respectively. However, the binding of the antibodies and antigens *in vivo* always needs several hours (even 24 h)^34^, and the blood flow would reduce the adhesion of synergist to the target area. In addition, there is a random connection between the Fc or Fab fragment and the synergistic agents^35^. Based on above mentioned reasons, the direct targeting is not satisfactory. Meanwhile, the clinical application of targeting synergists has been limited due to the difficult preparation. Pre-targeting technique has been originally developed for the radionuclide imaging and radioimmunotherapy, which separates the targeting molecules (antibodies) from the effector molecules (radionuclides). The targeting molecules are injected into the body first, and the effector molecules are injected at the peak concentration of the targeting molecules in the target tissue^36^. In the two-step biotin-avidin system pre-targeting technique, the binding between the antibodies and antigens *in vivo* is replaced by the binding between the biotin and avidin^37,38^. The affinity between biotin and avidin (KA: 10^15^ L/mol) is, at least, 10,000 times stronger than that between antigen and antibody (KA: 10^5–11^ L/mol), which could immediately form stable binding complex under physiological conditions. On the other hand, the four independent biotin-binding sites in avidin would allow for the amplification of the bio-signals, which could improve the targeting ability of the effector molecules^39–41^. Moreover, the biotin-avidin system is able to link different biotinylated antibodies to the same avidin labeled effector molecule^42^, and therefore the one effector molecule could be used in the treatment of various diseases. So, the biotin-avidin system has been characterized by the high sensitivity, high specificity, high stability, and wide adaptability. This technique has not only achieved satisfactory results in the radionuclide imaging and radioimmunotherapy^43^, but also been applied in other fields of imaging, such as MRI^44^ and ultrasound imaging^45^. Furthermore, in this study, the pre-targeting technique has been applied in the construction of the targeting synergistic agents for HIFU ablation. Streptavidin is a derivative of avidin, which has all the advantages while less disadvantages of avidin^46^. Thus, streptavidin is used to label the synergistic agents.

Malignant tumors are a major category of diseases threatening the lives and health of all human beings. Angiogenesis is closely related to tumor growth and invasion^47^. The vascular endothelial growth factor receptor-2 (VEGFR2) is highly expressed in the newly formed tumor blood vessels, which binds to VEGF to promote the angiogenesis^48^. Therefore, the VEGFR2 antibody was used as the targeting molecule.

In this study, we first prepared the HMME+PFP/PLGA nanoparticles and then modified them with the streptavidin bycarbodiimide method (HMME+PFP/PLGA-SA). Next, we analyzed the characteristics of HMME+PFP/PLGA-SA, including the particle size, entrapment efficiency, phase transition, and targeting ability, *in vitro*. Finally, we observed the therapeutic effect of HMME+PFP/PLGA-SA in HIFU ablation, targeting with two-step biotin-avidin pre-targeting technique, and explored the mechanisms underlying the synergistic effects.

## Results

### Characterization of HMME+PFP/PLGA-SA nanoparticles

**Figure 1:**
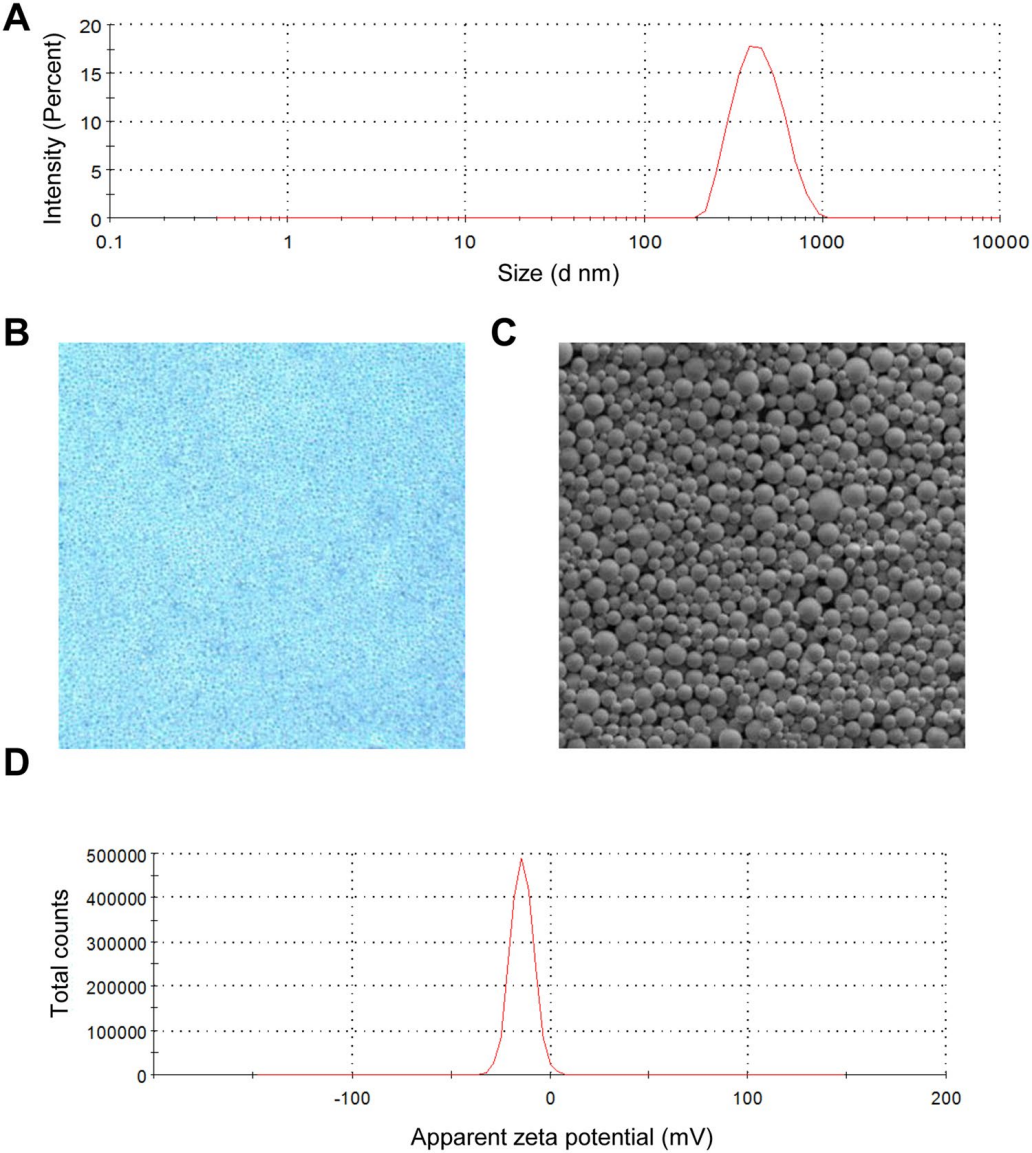
Characterization of HMME+PFP/PLGA-SA nanoparticles. (**A**) Diameter distribution. (**B**) HMME+PFP/PLGA-SA nanoparticles observed under light microscope (400×). (**C**) HMME+PFP/PLGA-SA nanoparticles under scanning electron microscopy (10000×). (**D**) Zeta potential distribution of HMME+PFP/PLGA-SA nanoparticles.

HMME+PFP/PLGA-SA nanoparticles were successfully prepared. The average particle size of the HMME+PFP/PLGA-SA nanoparticles was 477.8 ± 81.8 nm, ranging from 389.3 to 584.7 nm (Fig. 1A). These spherical nanoparticles were well dispersed, with uniform size and smooth surface (Fig. 1B,C). The Zeta potential was 13.9 ± 0.5 mV (Fig. 1D). The encapsulation efficiency of HMME and PFP were 46.6 ± 3.3% and 40.1 ± 2.6%, respectively.

### Phase transition of HMME+PFP/PLGA-SA nanoparticles

**Figure 2:**
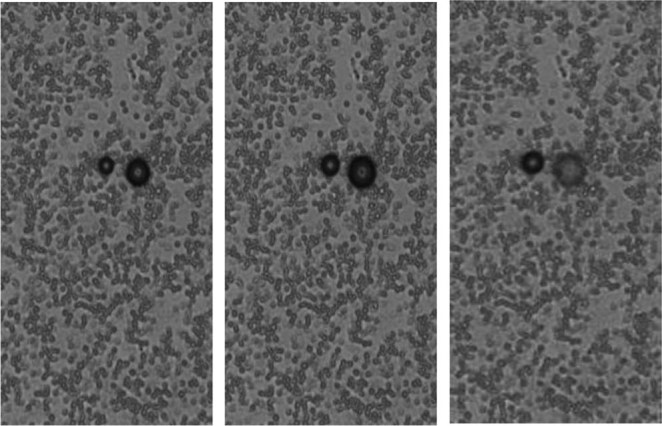
Phase transition of HMME+PFP/PLGA-SA at 45 °C (400×). At 45 °C, some nanoparticles became larger, gradually turned into microbubbles and finally broke.

**Figure 3:**
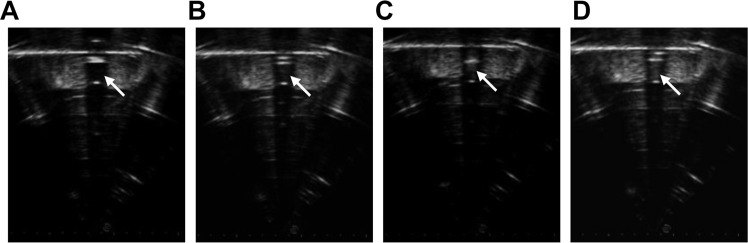
US images for HMME+PFP/PLGA-SA nanoparticles before and after HIFU treatment with different parameters. (**A**) US images for HMME+PFP/PLGA-SA nanoparticles before HIFU treatment. (**B–D**) US images for HMME+PFP/PLGA-SA nanoparticles after HIFU treatment with different parameters (**B**, 90 W and 5 s; **C**, 120 W and 5 s, and **D**, 150 W and 5 s). Before the HIFU irradiation, the HMME-PFP/PLGA-SA nanoparticles in the centrifugation tube showed no echo. After the HIFU irradiation, the echo increased obviously, and more obvious changes were observed along with the increasing power.

HMME+PFP/PLGA-SA nanoparticles were stable at room temperature. When the temperature was above 45 °C, or during the HIFU irradiation, phase transition can be induced. Our results showed that, when the temperature was set at 29 °C and 37 °C, no significant differences were observed in the particle size and morphology of HMME+PFP/PLGA-SA nanoparticles. However, at the temperature of 45 °C, some of the nanoparticles became larger and gradually turned into microbubbles, which finally broke. When the temperature was increased to 60 °C, more bubbles were generated, which quickly cracked. The number of bubbles at 60 °C (35.3 ± 6.5/HP) was significantly higher than that at 45 °C (18.0 ± 4.4/HP) (P < 0.05). Meanwhile, the residual nanoparticles were gradually decreased with the elongated duration (Fig. 2). Before the HIFU irradiation, the HMME-PFP/PLGA-SA nanoparticles in the centrifugation tube showed no echo. After the HIFU irradiation, the echo increased obviously, and more obvious changes were observed along with the increasing power (Fig. 3). The gray-scale changes of the nanoparticles with the output power of 90 W, 120 W, and 150 W were 17.0 ± 2.4, 32.0 ± 3.9, 45.4 ± 3.3, respectively.

### Binding between streptavidin and nanoparticles

**Figure 4:**
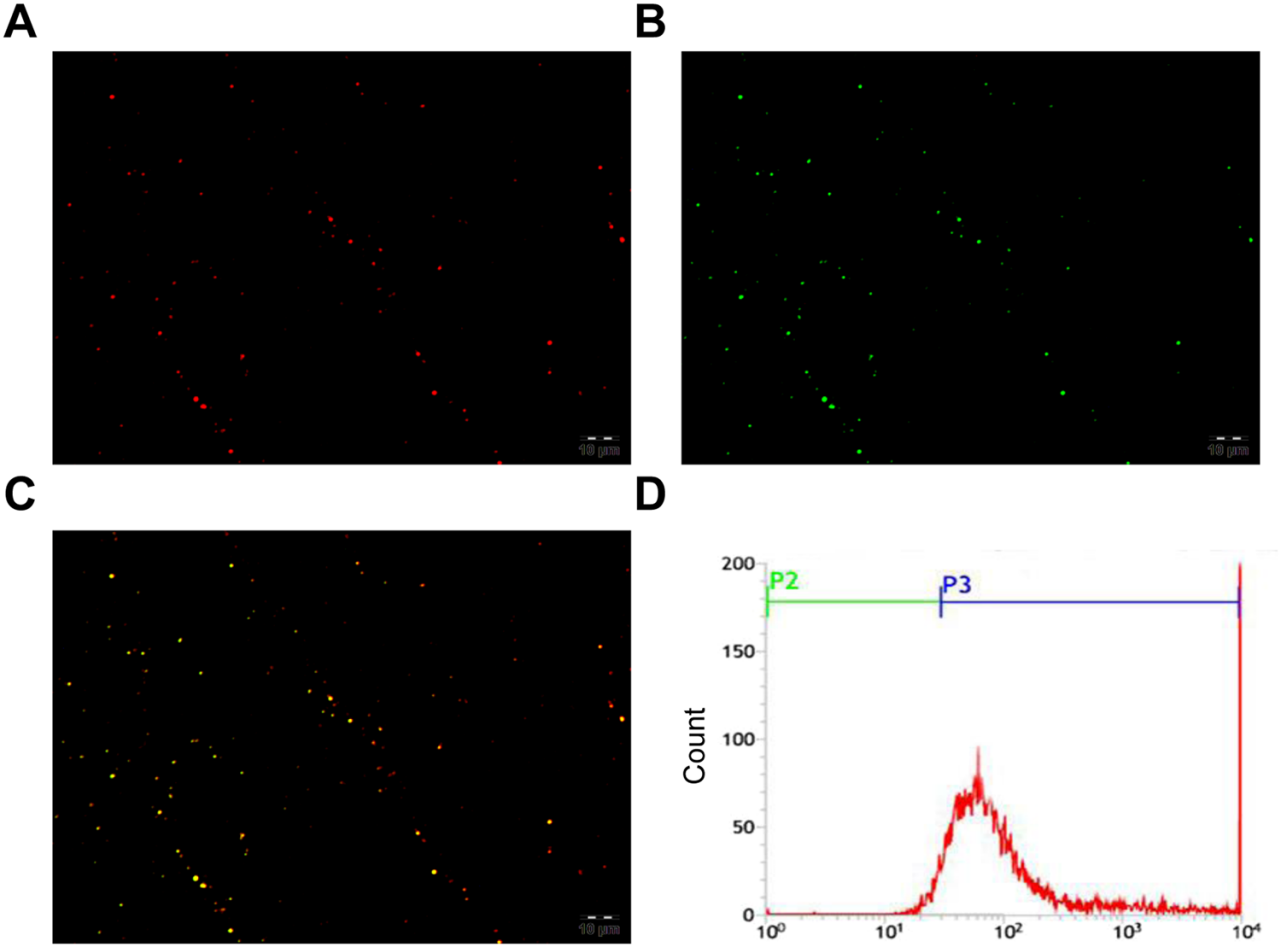
Detection of binding between streptavidin and nanoparticles. (**A**) Streptavidin-modified HMME+PFP/PLGA-SA nanoparticles were observed by CLSM (600×). CLSM showed red fluorescence from the streptavidin-modified hemoporphyrin monomethyl ether-loading liquid fluorocarbon (HMME+PFP/PLGA-SA) nanoparticle shell. (**B**) CLSM picture for the FITC-labeled streptavidin-modified HMME+PFP/PLGA-SA nanoparticles (600×). FITC-labeled streptavidin showed green fluorescence. (**C**) Merging image (600×). Yellow fluorescence was observed when emerging. (**D**) Binding between streptavidin and nanoparticles was detected by flow cytometry. The connection rate between streptavidin and liquid fluorocarbon nanoparticles was 95.5 ± 2.5%.

The streptavidin nanoparticle was a crucial step; therefore the binding between nanoparticles and streptavidin was assessed. Our results from the CLSM showed red fluorescence in the HMME+PFP/PLGA-SA nanoparticle shell (Fig. 4A). Moreover, FITC-labeled streptavidin showed green fluorescence (Fig. 4B), and the yellow fluorescence was observed when merged (Fig. 4C). These results suggest that the streptavidin is successfully connected to the nanoparticle surface. The flow cytometry showed that the connection rate between streptavidin and liquid fluorocarbon nanoparticles was very high (95.5 ± 2.5%) (Fig. 4D).

### Binding between HMME+PFP/PLGA-SA nanoparticles and targeted cells under flowing state

**Figure 5:**
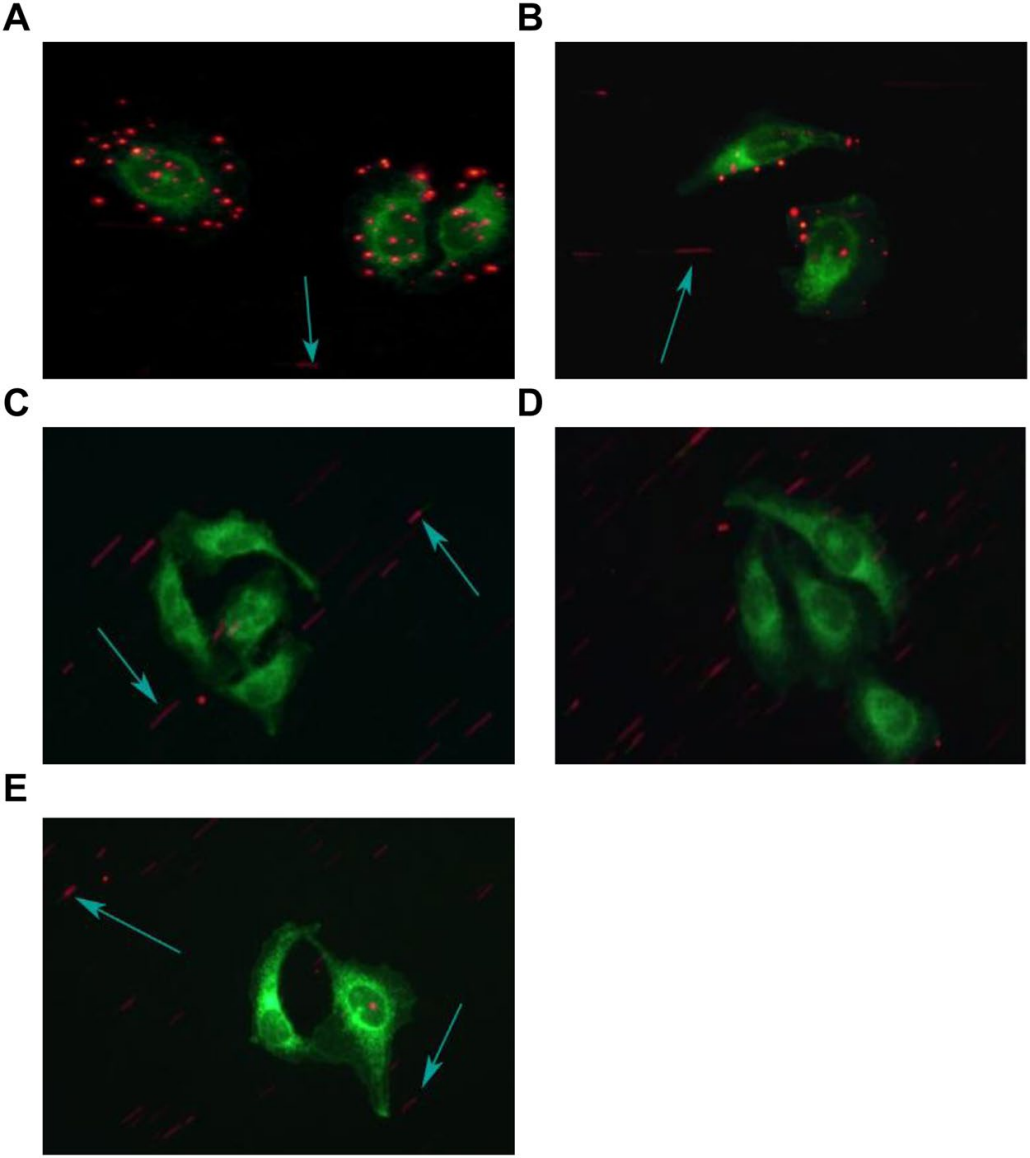
Detection of the combination between the nanoparticles and the cells (600×). Parallel plate flowing chamber test was performed to detect the combination between the nanoparticles and HUVECs. (**A**) The pre-targeting (HMME+PFP/PLGA-SA) group. (**B**) The direct targeting (HMME+PFP/PLGA-Ab) group. (**C**) The HMME+PFP/PLGA-SA group. (**D**) The HMM+PFP/PLGA group. (**E**) The antibody blocking group. In the pre-targeting (HMME+PFP/PLGA-SA) group, there were large amounts of nanoparticles binding to the cell surface and accumulating around the cells. In the direct targeting (HMME+PFP/PLGA-Ab) group, relatively few nanoparticles adhering to the cell surface were observed. For the HMME/PLGA, PFP/PLGA, and antibody blocking groups, there were almost no nanoparticles binding to the cell surface.

The binding between the HMME+PFP/PLGA-SA nanoparticles and HUVECs under flowing state was assessed with the parallel plate flowing chamber method. Our results showed that, in the pre-targeting (HMME+PFP/PLGA-SA) group, there were large amount of nanoparticles binding to the cell surface and accumulating around the cells. Moreover, in the direct targeting (HMME+PFP/PLGA-Ab) group, relatively few nanoparticles adhered to the cell surface. However, in the HMME/PLGA, PFP/PLGA, and antibody blocking groups, there were almost no nanoparticles binding to the cell surface (Fig. 5). Significant differences were observed in the amount of nanoparticles binding to one cell between the pre-targeting group and other groups (*P* < 0.05) (Table 1).

**Table 1:** Average number of adhering nanoparticles per cell in each group.

Group	Average adhering nanoparticle number
Pre-targeting	22.7 ± 3.1*
Direct targeting	8.1 ± 2.2
HMME+PFP/PLGA-SA	1.3 ± 1.2
HMME+PFP/PLGA	1.0 ± 0.7
Antibody blocking	0.6 ± 0.8

Note: Compared with other groups, *P < 0.05.

### Gray-scale changes and coagulation necrosis volume of tumors in nude mice after HIFU irradiation, and energy efficiency factor (EEF) value

**Figure 6:**
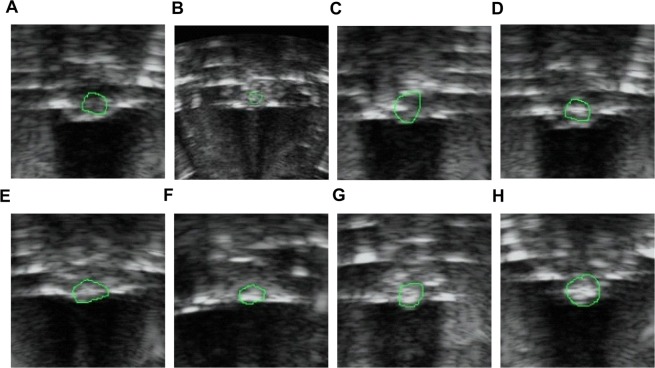
Gray-scale changes of targeted tissues before and after HIFU treatment. (**A**) Before and after HIFU treatment. (**B**) HIFU. (**C**) PLGA+HIFU. (**D**) PFP/PLGA+HIFU. (**E**) HMME/PLGA+HIFU. (**F**) HMME+PFP/PLGA+HIFU. (**G**) HMME+PFP/PLGA-Ab+HIFU. (**H**) HMME+PFP/PLGA-SA+HIFU. The tumor showed low echo in ultrasonography before HIFU treatment, and the gray-scale of the tumor target tissue area in all groups changed in varying degrees after the HIFU treatment.

Gray-scale changes, coagulation necrosis volume, and EEF value were used to evaluate the effects of HIFU after treatment. After the HIFU treatment, the gray-scale of the tumor target tissue area in all the groups changed in varying degrees. The most significant change was observed in the HMME+PFP/PLGA-SA+HIFU group, which was statistically different from other groups (*P* < 0.05). The gray-scale change in the HMME+PFP/PLGA-Ab+HIFU group was the second highest, with statistical significance compared with the other groups (*P* < 0.05). In addition, the gray-scale change in the HIFU group was significantly lower than other treatment groups (*P* < 0.05) (Table 2 and Fig. 6).

**Table 2:** Gray-scale changes before and after HIFU treatment.

Group	Gray-scale changes
HIFU	11.1 ± 1.9
PLGA+HIFU	16.9 ± 2.9*
PFP/PLGA+HIFU	29.6 ± 3.1*
HMME/PLGA+HIFU	25.5 ± 2.2*
HMME+PFP/PLGA+HIFU	34.9 ± 2.7*
HMME+PFP/PLGA-Ab+HIFU	44.7 ± 3.5*^#^
HMME+PFP/PLGA-SA+HIFU	61.3 ± 4.1*^#^

Note: Compared with the HIFU group, *P < 0.05; compared with the other groups, ^#^*P* < 0.05.

**Figure 7:**
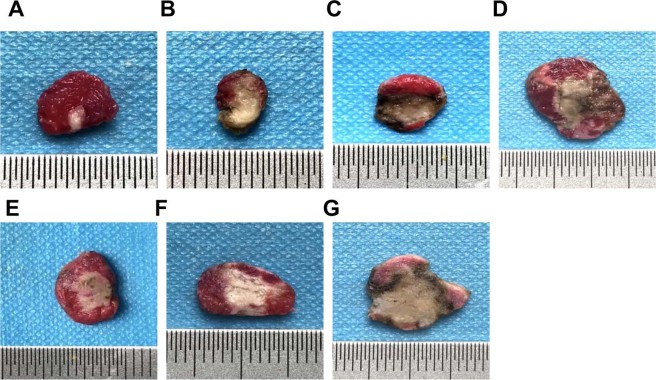
Detection of tumor coagulation necrosis volume after HIFU treatment. Tumor coagulation necrosis volume after HIFU treatment was detected with the TTC staining. (**A**) HIFU. (**B**) PLGA+HIFU. (**C**) PFP/PLGA+HIFU. (**D**) HMME/PLGA+HIFU. (**E**) HMME+PFP/PLGA+HIFU. (**F**) HMME+PFP/PLGA-Ab+HIFU. (**G**) HMME+PFP/PLGA-SA+HIFU. After the TTC staining, normal tissue was stained red, and the coagulation necrosis area was not stained (showing gray white).

In the TTC staining, the normal tissue was red, and the coagulation necrosis area was not stained (showing gray white). The coagulation necrosis volumes were measured and calculated. Our results showed that the largest coagulation necrosis volume was observed in the pre-targeting (HMME+PFP/PLGA-SA+HIFU) group, which was significantly different from other groups (*P* < 0.05), followed by the direct targeting (HMME+PFP/PLGA-Ab+HIFU) group. The smallest coagulation necrosis volume was observed in the HIFU group, which was statistically significant compared with other groups (*P* < 0.05) (Table 3 and Fig. 7).

**Table 3:** Coagulation necrosis volume and EEF before and after HIFU treatment.

Group	Coagulation necrosis volume	EEF
HIFU	8.7 ± 1.9	63.4 ± 15.0
PLGA+HIFU	14.7 ± 2.4*	36.6 ± 7.2*
PFP/PLGA+HIFU	28.6 ± 3.2*	18.6 ± 2.2^*^
HMME/PLGA+HIFU	27.5 ± 2.9*	19.3 ± 2.0*
HMME+PFP/PLGA+HIFU	41.6 ± 4.7*	12.8 ± 1.3*
HMME+PFP/PLGA-Ab+HIFU	53.7 ± 5.2*^#^	9.9 ± 1.0*
HMME+PFP/PLGA-SA+HIFU	79.1 ± 9.2*^#^	6.7 ± 0.7*^#^

Note: Compared with the HIFU group, *P < 0.05; compared with the other groups, ^#^*P* < 0.05.

**Figure 8:**
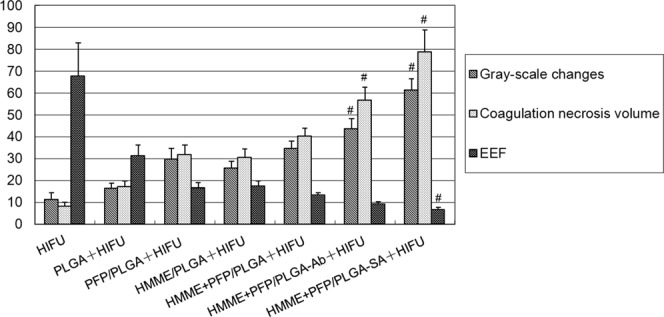
Histogram of gray-scale changes, coagulative necrosis volume, and EEF in tumor target tissue area after HIFU treatment. In the pre-targeting (HMME+PFP+SA/PLGA+HIFU) group, the gray-scale changes and coagulation necrosis volume of the tumor target area were significantly higher and enlarged, and had the lowest EEF value, which was statistically significant compared with other groups (*P* < 0.05).

On the other hand, similar results were observed for the EEF values. The lowest EEF value was observed in the pre-targeting (HMME+PFP/PLGA-SA+HIFU) group, which was statistically significant compared with the other groups (*P* < 0.05) (Table 3 and Fig. 8).

### Morphological changes by HE staining

**Figure 9:**
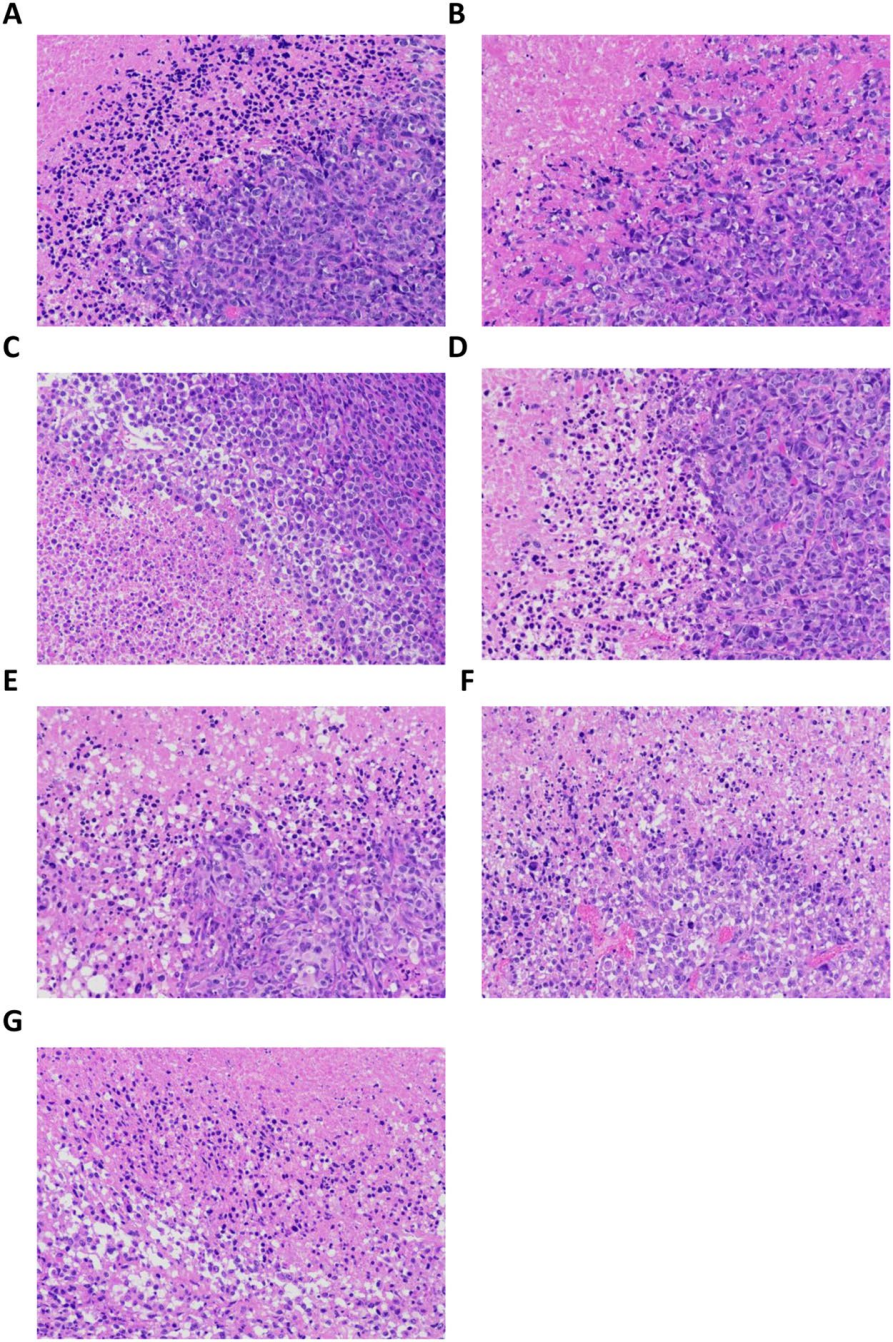
HE staining for the tumor tissues. (**A**) HIFU. (**B**) PLGA+HIFU. (**C**) PFP/PLGA+HIFU. (**D**) HMME/PLGA+HIFU. (**E**) HMME+PFP/PLGA+HIFU. (**F**) HMME+PFP/PLGA-Ab+HIFU. (**G**) HMME+PFP/PLGA-SA+HIFU. In the HIFU group, the tumor cells surrounding the coagulation necrosis area were intact in morphology and normally arranged. In the remaining treatment groups, damages were noted in the tumor cells surrounding the coagulation necrosis area, with nuclear condensation and sparsely arranged structure, as well as the homogenous red unstructured area (the vacuolar changes were occasionally seen). The most obvious changes were observed in the HMME+PFP/PLGA-SA+HIFU group.

Our results from the HE staining showed that the cellular structure of the coagulation necrosis area disappeared in each treatment group, showing red and unstructured area. In the HIFU group, the tumor cells surrounding the coagulation necrosis area were intact in morphology and were normally arranged. Moreover, in the remaining treatment groups, damages were observed in the tumor cells surrounding the coagulation necrosis area, with nuclear condensation and sparsely arranged structure, as well as the homogenous red unstructured area (the vacuolar changes were occasionally seen). The most obvious changes were observed in the pre-targeting (HMME+PFP/PLGA-SA+HIFU) group, with relatively obvious changes in the direct targeting (HMME+PFP/PLGA-Ab+HIFU) and HMME+PFP/PLGA+HIFU groups (Fig. 9).

### Results from CD34 staining, PCNA staining, and TUNEL staining

**Figure 10:**
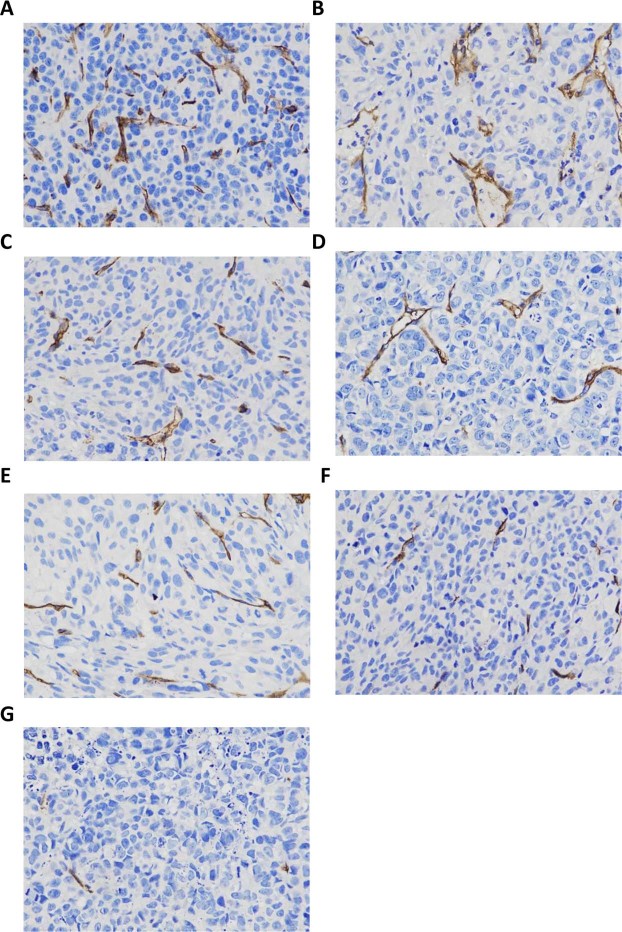
Expression of CD34 in tumors for different groups. (**A**) HIFU. (**B**) PLGA+HIFU. (**C**) PFP/PLGA+HIFU. (**D**) HMME/PLGA+HIFU. (**E**) HMME+PFP/PLGA+HIFU. (**F**) HMME+PFP/PLGA-Ab+HIFU. (**G**) HMME+PFP/PLGA-SA+HIFU. Neovessels were stained brown. There were less neovessels in the HMME+PFP/PLGA-SA+HIFU group. The number of neovessels was largest in the HIFU group.

CD34 staining by immunohistochemistry was used to observe the neovascularization in the tumor. Furthermore, proliferating cell nuclear antigen (PCNA) staining was used to evaluate the proliferation of cells and the TdT-mediated dUTP Nick-End Labeling (TUNEL) staining was performed to evaluate cellular apoptosis. Immunohistochemistry showed that CD34 was localized in the cytoplasm or on the membrane of vascular endothelial cells, and neovessels were stained brown. The neovessels were counted under high magnification and microvessel density (MVD) was calculated. Our results showed that, compared with the other groups, there were significantly less neovessels in the pre-targeting (HMME+PFP/PLGA-SA+HIFU) group (8.3 ± 1.5/HP; compared with other groups, *P* < 0.05). The number of neovessels was the largest in the HIFU group, significantly higher than other groups (*P* < 0.05) (Table 4 and Fig. 10).

**Table 4:** Comparison of MVD of tumor tissues (count/HP).

Group	MVD
HIFU	56.7 ± 7.8
PLGA+HIFU	38.5 ± 5.5*
PFP/PLGA+HIFU	26.6 ± 4.5*
HMME/PLGA+HIFU	24.9 ± 4.7*
HMME+PFP /PLGA+HIFU	20.5 ± 3.7*
HMME+PFP/PLGA-Ab+HIFU	15.1 ± 2.5*
HMME+PFP/PLGA-SA+HIFU	8.3 ± 1.5*^#^

Note: Compared with the HIFU group, *P < 0.05; compared with the other groups, ^#^*P* < 0.05.

**Figure 11:**
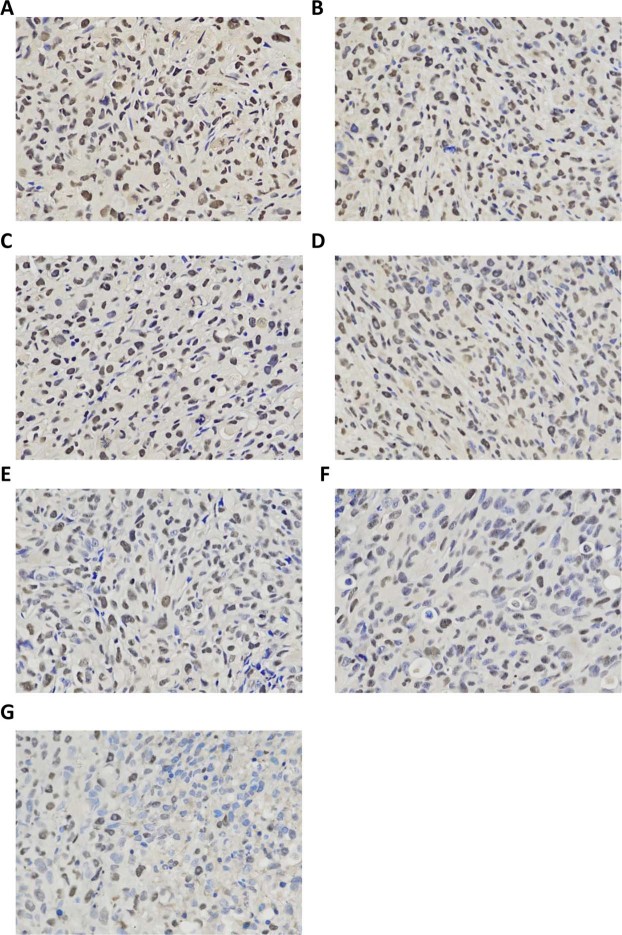
Expression of PCNA in tumors for different groups. (**A**) HIFU. (**B**) PLGA+HIFU. (**C**) PFP/PLGA+HIFU. (**D**) HMME/PLGA+HIFU. (**E**) HMME+PFP/PLGA+HIFU. (**F**) HMME+PFP/PLGA-Ab+HIFU. (**G**) HMME+PFP/PLGA-SA+HIFU. PCNA staining showed brown staining particles with different degrees in the nucleus for each group. The cells with the weakest staining were noted for the pre-targeting (HMME+PFP/PLGA-SA+HIFU) group, followed by the direct targeting (HMME+PFP/PLGA-Ab+HIFU) group. The cells with the strongest staining were observed in the HIFU group.

PCNA staining showed that brown particles with different staining degrees were observed in the nucleus. The cells with the weakest staining were noted in the pre-targeting (HMME+PFP/PLGA-SA+HIFU) group, followed by the direct targeting (HMME+PFP/PLGA-Ab+HIFU) group. The cells with the strongest staining were observed in the HIFU group (Fig. 11). Compared with the HIFU group, proliferation indexes (PIs) in all the other groups were statistically significant (*P* < 0.05). The PI of the pre-targeting group was statistically significant compared with the other groups (*P* < 0.05) (Table 5).

**Table 5:** PI for tumor tissue.

Group	PI
HIFU	93.4 ± 3.7
PLGA+HIFU	86.9 ± 3.9*
PFP/PLGA+HIFU	83.5 ± 3.7*
HMME/PLGA+HIFU	79.2 ± 3.1*
HMME+PFP/PLGA+HIFU	68.2 ± 2.5*
HMME+PFP/PLGA-Ab+HIFU	59.1 ± 3.7*
HMME+PFP/PLGA-SA+HIFU	45.9 ± 4.8*^#^

Note: Compared with the HIFU group, *P < 0.05; compared with the other groups, ^#^*P* < 0.05.

**Figure 12:**
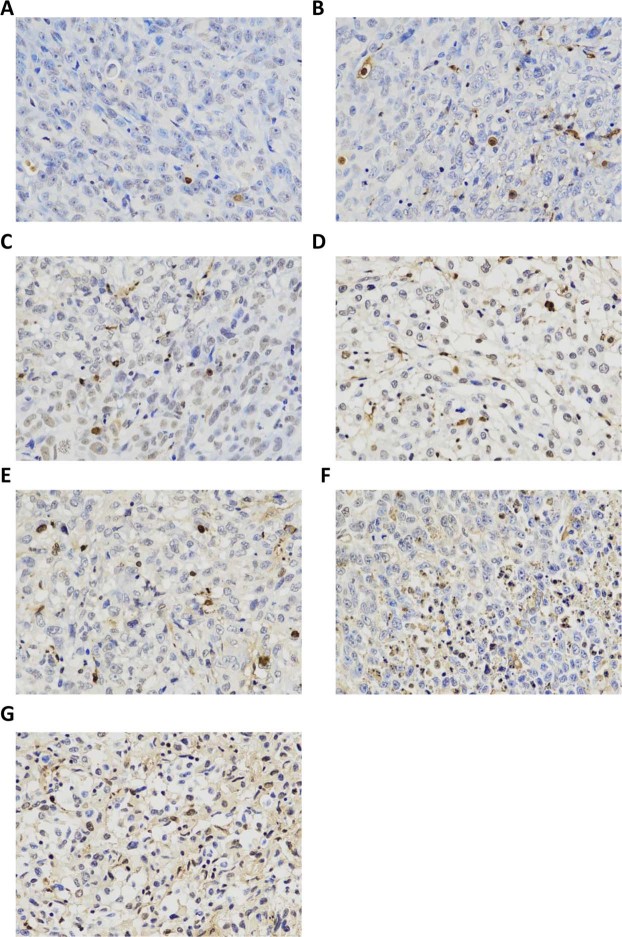
Apoptosis in tumors for different groups. TUNEL detection was performed to assess the apoptosis in tumors. (**A**) HIFU. (**B**) PLGA+HIFU. (**C**) PFP/PLGA+HIFU. (**D**) HMME/PLGA+HIFU. (**E**) HMME+PFP/PLGA+HIFU. (**F**) HMME+PFP/PLGA-Ab+HIFU. (**G**) HMME+PFP/PLGA-SA+HIFU. The TUNEL staining showed that brown staining with different degrees in the nucleus for each group. The cells with the strongest staining were noted for the pre-targeting (HMME+PFP/PLGA-SA+HIFU) group, followed by the direct targeting (HMME+PFP/PLGA-Ab+HIFU) group. The cells with the weakest staining were observed in the HIFU group.

TUNEL staining showed that brown staining with different degrees was observed in the nucleus. The cells with the strongest staining were noted in the pre-targeting (HMME+PFP/PLGA-SA+HIFU) group, followed by the direct targeting (HMME+PFP/PLGA-Ab+HIFU) group. The cells with the weakest staining were observed in the HIFU group (Fig. 12). Compared with the HIFU group, apoptotic indexes (AIs) in all the other groups were statistically significant (*P* < 0.05). Compared with the other groups, the AIs of the pre-targeting group and the direct targeting group were statistically significant (*P* < 0.05) (Table 6).

**Table 6:** AI for tumor tissue.

Group	AI
HIFU	6.6 ± 1.7
PLGA+HIFU	12.9 ± 2.6*
PFP/PLGA+HIFU	16.8 ± 3.0*
HMME/PLGA+HIFU	20.5 ± 3.3*
HMME+PFP /PLGA+HIFU	35.6 ± 3.4*
HMME+PFP/PLGA-Ab+HIFU	43.3 ± 4.0*^#^
HMME+PFP/PLGA-SA+HIFU	59.1 ± 4.7*^#^

Note: Compared with the HIFU group, *P < 0.05; compared with the other groups, ^#^*P* < 0.05.

**Figure 13:**
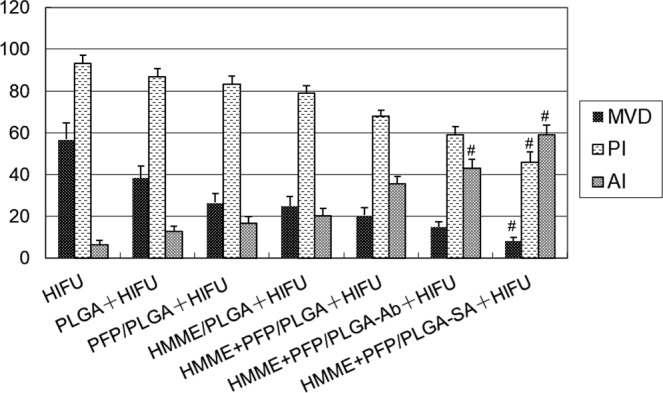
Histogram of MVD, PI, and AI in tumor tissue around the coagulation necrosis area after HIFU treatment. The MVD and PI in the tumor tissue around the coagulation necrosis area in the pre-targeting group were significantly reduced while the AI were significantly increased compared with other groups (*P* < 0.05).

In brief, the MVD and PI were declined, while the AI was increased, in the tumor tissue surrounding the coagulation necrosis area of the pre-targeting group (Fig. 13).

### Assessment of tumor growth rate and survival time of tumor-bearing nude mice

**Figure 14:**
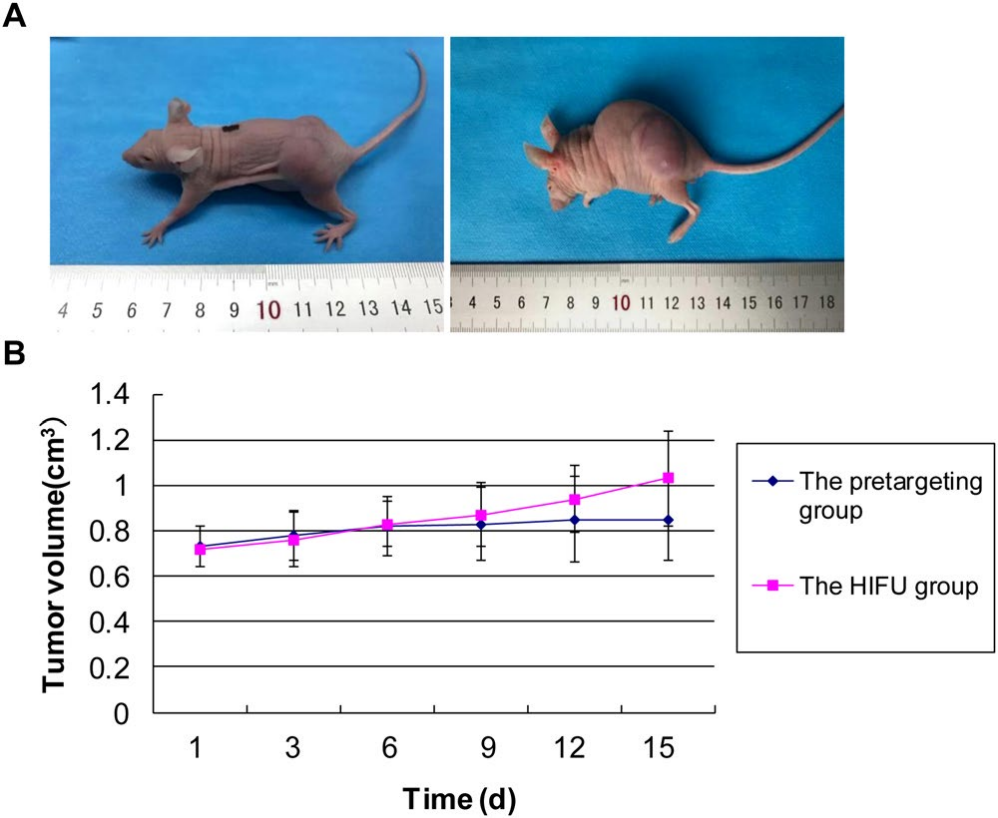
Tumor volume curve before and after HIFU treatment. (**A**) Tumor-bearing nude mice before and after HIFU treatment. Left, before the HIFU treatment; and right, after the HIFU treatment, there were no scald lesions on the skin. (**B**) Tumor volume curves. There was no significant difference in the tumor volume between the groups before treatment. After the treatment, relatively slower tumor growth rate was observed for the pre-targeting group. At day 15 after treatment, there were significant differences in the tumor volume between the pre-targeting and HIFU groups.

There was no significant difference in the tumor volume between the groups before treatment (*P* > 0.05). The tumor volume and growth curves were shown in Table 7 and Fig. 14. After the HIFU treatment, there were no scald lesions on the skin of the nude mice (Fig. 14A). Our results showed that, after the treatment, relatively slower tumor growth rate was observed in the pre-targeting group. At day 15 after treatment, there were significant differences in the tumor volume between the pre-targeting group and HIFU group (*P* < 0.05) (Fig. 14B). The general condition of all mice was good in the early stage, but gradually became cachexia, ending up with death. The longest survival time of the tumor-bearing nude mice was 24 days in the pre-targeting group, with the average survival time of 16.2 ± 5.8 days. However, the longest survival time in the HIFU group was 18 days, with the average survival time of 11.7 ± 6.3 days. Significant difference was observed in the survival time between these two groups (*P* < 0.05).

**Table 7:** Tumor volume at different times before and after treatment (cm^3^).

	Days after treatment (d)					
	0	3	6	9	12	15
HIFU	0.72 ± 0.07	0.76 ± 0.09	0.84 ± 0.10	0.88 ± 0.09	0.94 ± 0.10	1.01 ± 0.10
Pre-targeting	0.73 ± 0.07*	0.76 ± 0.06	0.82 ± 0.07	0.83 ± 0.09	0.84 ± 0.07	0.86 ± 0.10^#^

Note: Compared with the HIFU group, *P > 0.05; compared with the HIFU group, ^#^*P* < 0.05.

## Discussion

In this study, PLGA was used as film-forming material, and single emulsification method was applied to encapsulate HMME and PFP to prepare liquid fluorocarbon (HMME+PFP/PLGA) nanoparticles. The carbodiimide method was used to combine the amino groups on the streptavidin with the carboxyl groups on the PLGA to form stable ester bonds, thereby producing streptavidin-modified nanoparticles (HMME+PFP/PLGA-SA). The average particle size of the HMME+PFP/PLGA-SA nanoparticles was 477.8 ± 81.8 nm, ranging from 389.3 to 584.7 nm. Our results showed that HMME+PFP/PLGA-SA remained stable at body temperature (37 °C), when the temperature rised above 45 °C, or with the HIFU irradiation, liquid-vapor phase transition was induced, which could be used as the cavitation nucleus in HIFU treatment. Danny *et al*. have prepared liposome nanoparticles containing PFP and Dox, and the nanoparticles were spherical at 37 °C, and they became deformed at 42 °C, in line with our observations.

Nanoparticle-based cancer diagnostics and therapeutics can be significantly enhanced by selective tissue localization, but the strategy can be complicated by the requirement of a targeting ligand conjugated on nanoparticles. The core premise of pre-targeting lies in administering the targeting ligand and nanoparticles separately and having the two components combine *in vivo*. Pre-targeting approaches based on biotin and streptavidin are among the earliest strategies to emerge and the first to be applied in the clinic^49–51^.Yang *et al*.^42^ have designed streptavidin-scFv fusion proteins and biotinylated nanoparticles for targeting lymphoma cells through pre-targeting strategy. Their results demonstrated that greater uptake was observed for biotinylated nanoparticles, with 6- to 18-fold higher uptake vs. non-biotinylated nanoparticle and fusion protein controls. In our study, the pre-targeting (HMME+PFP/PLGA-SA) group showed a large number of nanoparticles binding to the cellular surface in the flowing state, and the nanoparticles based on pre-targeting technology had strong targeting ability, which provides the foundation for the *in vivo* targeting experiments. In this study, the pre-targeting technology was used to enhance the targeting of synergistic agent in HIFU, which is of great significance. The synergistic agent could adhere to the target area more efficiently and quickly, thus increasing the concentration of local synergistic agent. Moreover, one targeted synergistic agent can be used for various tissues.

In the *in vivo* pre-targeting enhanced HIFU ablation, the gray-scale changes and coagulation necrosis volume of the tumor target area in the pre-targeting (HMME+PFP/PLGA-SA+HIFU) group were significantly higher and enlarged than the direct target group and other groups. HE staining showed that damages were observed in tumor cells surrounding the coagulation necrosis area, with nuclear condensation and sparsely arranged structure, as well as red unstructured regions (vacuolar changes were seen), especially in the pre-targeting (HMME+PFP/PLGA-SA+HIFU) group. And, the pre-targeting group had significantly reduced microvessel density in the tumor tissue around the coagulation necrosis area and had the lowest expression of PCNA in the tumor cells, suggesting that the proliferation of tumor cells is effectively inhibited. Meanwhile, the apoptotic cells were significantly increased in the tumor tissue around the coagulation necrosis. The mechanism might be that, HMME+PFP/PLGA-SA is nanoscale, which could penetrate the tumor vascular endothelium into the interstitial space. The biotinylated VEGFR2 antibodies was pre-administered and heavily adhered to the neovascular endothelial cells with high receptor expression. When the HMME+PFP/PLGA-SA was added, the high-affinity biotin-avidin system could rapidly and stably combine with the biotinylated VEGFR2 antibody, increasing the number of nanoparticles in the tumor tissue. During HIFU irradiation, phase transition of the liquid fluorocarbon (PFP) encapsulated in the nanoparticles would occur when the temperature increased above 45 °C. The phase transition could increase the cavitation effects of HIFU, which in turn generates high-temperature environment, increases the accumulation of acoustic energy, and activates the HMME^52^. Moreover, active substances could be generated from the activated HMME^53,54^. Therefore, the cavitation effect of HIFU and sonodynamic therapy could exert synergistic effects. At the same time, the VEGFR2 antibody, which acted as targeting molecule and directly bound to the VEGFR2 receptor, may be block the angiogenic signal pathway and reduce the formation of new blood vessels. The characteristic of the synergistic agent is that it could induce phase transition and produce bubbles in the tissue surrounding coagulation necrosis areas (the temperature ranges from 45 °C to 60 °C), thereby loosing the tissue structure^55,56^. Meanwhile, the cytotoxic substances produced by the sonosensitizer could enter the tissues. In addition, the VEGFR2 antibody may inhibit neovascularization. These synergistic effects could induce secondary necrosis in the tissue surrounding the HIFU-induced coagulation necrosis area. These synergistic agents are very suitable for ablation of deep tumor or tumors with barrier along the acoustic beam path, and other areas of difficult ablation. Because conventional HIFU ablation often causes damage on residual tissues, the secondary effects induced by the synergistic agents could kill these residual cells.

Despite these findings, yet both the immunogenicity of streptavidin and the presence of endogenous biotin have proven complicating factors^49^. We expect that the application of alternative binding pairs would avoid the problematic, development of pre-targeting molecules with high binding affinities to both cells and nanoparticles and bio-inert binding pairs (such as bioorthogonal chemical reactions between small molecule tags)^42,57^.

There are some limitations for this study. The *in vivo* distribution of HMME+PFP/PLGA-SA was not investigated. Further studies are still needed to analyze the accumulation in the targeting area in tumors with the *in vivo* fluorescence imaging. MR thermometry should also be used to investigate thermal deposition *in vivo*. Only the tumor volume and survival time of the tumor-bearing nude mice were investigated after the enhanced HIFU treatment. Further in-depth studies are still needed to analyze the tumor outcomes with ultrasound, MRI or pathology analysis.

In conclusion, this study explored a novel phase-shift nanoparticles and applied pre-targeting for enhanced HIFU ablation. The HMME+PFP/PLGA nanoparticles were prepared and then subjected to the streptavidin modification. The two-step biotin-avidin pre-targeting technique was applied to HIFU ablation *in vivo* with biotinylated VEGFR2 antibody. Our results showed that, the largest gray-scale changes and coagulation necrosis areas were observed in the pre-targeting (HMME+PFP/PLGA-SA) group, with the lowest EEF value. Moreover, the MVD and PI were declined, while the AI was increased, in the tumor tissue surrounding the coagulation necrosis area. Meanwhile, the survival time of the tumor-bearing nude mice of the pre-targeting group was significantly longer than the HIFU treatment group. These results suggest that HMME+PFP/PLGA-SA can effectively enhance the effects of HIFU ablation *in vivo* in a targeted manner, which may be used as a potential targeting synergist. The pre-targeting technology is introduced to the HIFU targeting ablation, providing novel idea for the development of targeting synergists for HIFU.

## Materials and Methods

### Preparation of streptavidin-modified hemoporphyrin monomethyl ether-loading liquid fluorocarbon (HMME+PFP/PLGA-SA) nanoparticles

HMME+PFP/PLGA-SA nanoparticles were prepared by the emulsification method^28^. Briefly, 25 mg PLGA (lactide:glycolide = 50:50; MW = 12000; Daigang, China) and 2 mg HMME(Shanghai D B Chemical Technology Co., Ltd., China) were dissolved in 2 mL trichloromethane (CHCl_3_). After adding 200 µL PFP (strem chemicals Inc., USA) and 8 mL poly (vinyl alcohol) (PVA, 4%, w/v, MW = 25,000, Sigma) solution, the mixture was subjected to acoustic vibration on ice (XL2020, Heat System, Inc, USA), with the power of 100 W; vibrating for 5 s, with 5-s intervals, for totally 6 min). The mixture was added with 10 mL 2% isopropanol, and then magnetically stirred for 3 h. After centrifugation (Eppendorf AG, Germany) at 8,000 rpm for 5 min, the HMME+PLGA/PFP nanoparticles were obtained. HMME+PFP/PLGA nanoparticles were dispersed in an appropriate amount of MES solution (0.1 M, pH 5.1). EDC and NHS were subsequently added (the mole ratio of PLGA-COOH to EDC was 1:10, and the mass ratio of EDC to NHS was 1:3), and the mixture was shaken on ice for 45 min. After washing with ddH_2_O, the nanoparticles were again dispersed in the MES solution (0.1 M, pH 8.0). After adding SA, the mixture was incubated on ice for 2 h. The HMME+PFP/PLGA-SA nanoparticles were obtained after washing with ddH_2_O. For the preparation of HMME+PFP/PLGA-Ab, the same procedures were performed, except that the SA was replaced by Ab.

### Characterization of HMME+PFP/PLGA-SA nanoparticles

The size and distribution of HMME+PFP/PLGA-SA nanoparticles were observed with the inverted fluorescence microscope (Olympus CKX41, Japan). The particle size and surface potential of these nanoparticles were detected with the Malvern Zetasizer Nano ZS (Malvern, UK). The morphological features were observed by the scanning electron microscopy (SEM, Hitachi S-3400N, Japan), and the fluorescence characteristics of these nanoparticles were observed by confocal laser scanning microscopy (CLSM, Leica TCS-SP2, German).

### Assessment of entrapment efficiency of HMME+PFP/PLGA-SA nanoparticles

After destruction with CHCl_3_ and centrifugation, the insoluble substance at the bottom was PFP. The PFP entrapment efficiency was obtained by dividing the PFP weight with the original weight (with the density of 1.63 g/mL). The nanoparticles were dissolved with DMSO, and the HMME entrapment efficiency was detected with the UV spectrophotometer (NanoDrop 2000, Thermo Scientific, UAS)^28^.

### Assessment of effects of increased temperature on HMME+PFP/PLGA-SA phase transition

The effects of increased temperature on HMME+PFP/PLGA-SA nanoparticle phase transition were assessed by the heating plate method. The diluted nanoparticles were dropped on a slide glass, which was placed on the heating plate. Along with the increasing temperature (29 °C, 37 °C, 45 °C, and 60 °C, respectively), the effects of increased temperature on the nanoparticle phase transition were observed with the microscope.

### Assessment of effects of HIFU irradiation on HMME+PFP/PLGA-SA phase transition

HIFU irradiation was performed using the JC-200 focused ultrasound system (Chongqing Haifu Technology, Chongqing, China) as described previously^58^. The system mainly consisted of therapeutic ultrasound unit, diagnostic ultrasound unit, and central processing system. The therapeutic transducer had the focal length of 145 mm, diameter of 220 mm, and working frequency of 0.94 MHz. The acoustic focal region was 11 mm along the beam axis and 3 mm in the transverse direction. Continuous-wave HIFU was applied in all the studies. The diagnostic transducer with center frequencies of 3.5–5 MHz was installed in the center of the therapeutic transducer, which moved together to guide and monitor the treatment procedure in real time. The integrated transducers were submerged in degassed water.

The diluted HMME+PFP/PLGA-SA nanoparticles (3 mL) were added into the centrifugation tube. The tube top was amounted onto the wooden plate, and the tube bottom was placed in the HIFU degassing tank. The therapeutic probe was adjusted to the center of the liquid in the tube, and the HIFU single point radiation was started, with the output power of 90 W, 120 W, and 150 W, the corresponding acoustic intensities were approximately 1286 W/cm^2^, 1714 W/cm^2^, and 2143 W/cm^2^, respectively. The irradiation time was 5 s. The gray levels of the liquid after radiation were observed.

### Assessment of streptavidin and nanoparticle binding

In the preparation of HMME+PFP/PLGA-SA, FITC-SA was used instead, to obtain the FITC-labeled nanoparticles (HMME+PFP/PLGA-SA-FITC). The binding between streptavidin and nanoparticles was detected with CLSM and flow cytometry (FACSVantage SE, BD, USA), respectively.

### Assessment of binding between HMME+PFP/PLGA-SA nanoparticles and cells under flowing state

The binding between the HMME+PFP/PLGA-SA nanoparticles and the cells under flowing state was assessed with the parallel plate flow chambers method (ClycoTech, USA). Human umbilical vein endothelial cells (HUVECs) were cultured with the DMEM medium containing 10% fetal bovine serum (FBS), supplemented with 1% streptomycin-penicillin, at the density of 1 × 10^5^ cells/dish, in a 37 °C, 5% CO_2_ incubator. After 24 h, cells were divided into the following groups: (1) the pre-targeting (HMME+PFP/PLGA-SA) group; (2) the direct targeting (HMME+PFP/PLGA-Ab) group; (3) the HMME+PFP/PLGA-SA group; (4) the HMME+PFP/PLGA group; and (5) the antibody blocking group. The pre-targeting group was pretreated and cultured with 100 μL biotinylated VEGFR2 (100 μg/mL, Bio-Techne, USA) for 24 h, while the antibody blocking group was cultured with 100 μL VEGFR2 (100 μg/mL, Bio-Techne, USA) for 24 h. For the assessment, the cell membrane was stained with DIO, and then the culture dish was connected to the parallel plate flow chambers, with flowing liquid at the rate of 100 μL/min. For all the groups, the cells were treated with 200 μL HMME+PFP/PLGA-SA, HMME+PFP/PLGA-Ab, HMME+PFP/PLGA-SA, HMME+PFP/PLGA, and HMME+PFP/PLGA-SA, respectively. After adding the nanoparticles, the groups were washed with PBS (at 100 μL/min for 15 min), and the binding of the nanoparticles to the cells was observed with the inverted fluorescence microscope (Olympus CKX41, Japan). Totally 10 fields (600×) were selected, and the cells bound with the nanoparticles were counted.

### Study animals

The BALB/c nude mice, 4–6 weeks old, weighing 20.3 ± 1.9 g, were purchased from the Animal Center of Chongqing Medical University (Chongqing, China). All animal experiments were carried out in accordance with the ethical guidelines of the Chongqing Medical University [Approval Number: SYXK (Chongqing) 2017–0023]. And all animal study protocols were approved by the Animal Care and Protection Committee at Chongqing Medical University. The mice were subcutaneously injected with the human breast cancer MDA-MB-231 cells (1 × 10^6^) at the buttocks. After 4–6 weeks, when the tumor diameter reached about 1.5 cm, the mice were ready for the experiments.

### Biotinylated VEGFR2 antibody-mediated pre-targeting of HMME+PFP/PLGA-SA nanoparticles for targeting enhanced HIFU ablation

Totally 70 tumor-bearing nude mice were randomly divided into the following 7 groups (n = 10): the HIFU, PLGA+HIFU, PFP/PLGA+HIFU, HMME/PLGA+HIFU, HMME+PFP/PLGA+HIFU, direct targeting (HMME+PFP/PLGA-Ab+HIFU), and pre-targeting (HMME+PFP/PLGA-SA+HIFU) groups. After anesthetizing with 1% pentobarbital sodium, the nude mouse was subjected to the single point HIFU radiation at the tumor site, with the following parameters: P 150 W and T 5 s, the acoustic intensity was approximately 2143 W/cm^2^. The mice from in HIFU group received HIFU irradiation alone, and the mice in the pre-targeting group were injected with 100 μL biotinylated VEGFR2 antibody (100 μg/mL) through the tail vein at 24 h before HIFU irradiation. Then the groups were treated with 200 μL of PLGA, PFP/PLGA, HMME/PLGA, HMME+PFP/PLGA, HMME+PFP/PLGA-Ab, and HMME+PFP/PLGA-SA, respectively. After 5 min, the mice received the HIFU treatment.

### Assessment of gray scales before and after irradiation

The changes in the gray scales in the tumor target region before and after irradiation were observed and analyzed by the same technician, with the software equipped on the HIFU instrument (Esaote MyLab, Italy) under the same parameter setting (3.5 MHz, power 75%, Gain 34%, Dyn range 12, Mechanical Index 0.6, Depth 17 cm).

### Assessment of coagulation necrosis volume

At 1 d after HIFU treatment, the nude mice were sacrificed, and the tumors were removed. The tumor was cut into the 1–2 mm section series, along the long axis of the sonic beam. Then the tissue was stained with 1% TTC solution (Sigma, USA) in 37 °C water bath for 30 min. The necrosis within the tumor was observed. The length, width, and thickness were measured (in mm^3^), and the necrosis volume was calculated accordingly (V = π/6 × length × width × thickness).

### Energy efficiency factor (EEF) evaluation

EEFs for nanoparticles under different parameters were calculated as follows: EEF (J/mm^3^) = ηPt/V^59^, where η was the HIFU transducer focus factor (which was 0.7 in this case), P (W) was the total output sound power, t (s) was the total treatment time, and V (mm^3^) was the coagulation necrosis volume.

### HE staining, CD34 staining, PCNA staining, and TUNEL detection

The coagulation necrosis area and adjacent tumor tissues were fixed with paraformaldehyde solution. HE staining, CD34 staining, PCNA staining, and TUNEL detection were performed to assess the tissue within 3 mm around the coagulation necrosis tissue. The CD34 positive staining was defined as the brown staining in the cytoplasm or on the membrane of vascular endothelial cells. The positive cells were counted, and the MVD was calculated^60^.

PCNA and TUNEL positive staining was defined as yellow or brown staining in the nucleus with higher intensity than the background. The positive cells in each section were counted under high-magnification microscope (400×), and the following indexes were calculated according to the following formula: for the PCNA staining, PI = number of positive cells/total number of cells × 100%; and for the TUNEL staining, AI = number of positive cells/total number of cells × 100%

### Assessment of tumor growth rate and survival time in tumor-bearing nude mice

The 12 tumor-bearing nude mice were randomly divided into the HIFU group and the HMME+PFP/PLGA-SA+HIFU (pre-targeting) group. The same therapies were performed as mentioned above. The tumor growth rate and survival time in tumor-bearing nude mice were assessed. After treatment, the long and short diameters of the tumor were measured every 3 days, and the tumor volume (TV) was calculated as follows: TV (mm^3^) = π/6 × length × width × thickness.

### Statistical analysis

Data were expressed as mean ± SD. SPSS 25.0 software was used for statistical analysis. Paired *t*-test was used for pair-wise comparison, and the analysis of variance was used for multiple group comparison. The data without normal distribution was analyzed with rank test. *P* < 0.05 was considered as statistically significant.

## Data Availability

The data that support the findings of this study are available on request from the corresponding author.
